# Sarcopenia Diagnosed Using Masseter Muscle Diameter as a Survival Correlate in Elderly Patients with Glioblastoma

**DOI:** 10.1016/j.wneu.2022.02.038

**Published:** 2022-02-15

**Authors:** Ramin A. Morshed, Jacob S. Young, Megan Casey, Elaina J. Wang, Manish K. Aghi, Mitchel S. Berger, Shawn L. Hervey-Jumper

**Affiliations:** 1 Department of Neurological Surgery, University of California, San Francisco, San Francisco, California; 2 Warren Alpert Medical School, Brown University, Providence, Rhode Island, USA

**Keywords:** Biopsy, Complications, Elderly, Masseter, Octogenarian, Resection, Sarcopenia

## Abstract

**BACKGROUND:**

Elderly patients with glioblastoma (GBM) have a worse prognosis than do younger patients. The present study aimed to identify the patient, treatment, and imaging features, including measures of sarcopenia, associated with worse survival and 90-day postoperative mortality for elderly patients with GBM.

**METHODS:**

A single-center retrospective study was conducted of patients aged ≥79 years at surgery who had undergone biopsy or resection of a World Health Organization grade IV GBM at the initial diagnosis. Imaging features of sarcopenia were collected, including the masseter and temporalis muscle diameters. Multivariate analyses were performed to identify factors associated with survival and 30-day complications.

**RESULTS:**

The cohort included 110 patients with a mean age of 82.8 years at surgery and a median preoperative Karnofsky performance scale score of 80. The majority of patients underwent a surgical resection (66.4%) while a minority underwent biopsy (33.6%). Adjuvant chemo- and/or radiation therapy were used in 72.5% of the cohort. On multivariate analysis, age (hazard ratio [HR], 7.97; 95% confidence interval [CI], 1.63–36.3), adjuvant therapy (RT or TMZ vs. none: HR, 0.12; 95% CI, 0.05–0.3; RT plus TMZ vs. none: HR, 0.05; 95% CI, 0.02–0.14), surgical resection (HR, 0.46; 95% CI, 0.24–0.9), multifocality (HR, 2.7; 95% CI, 1.14–6.4), and masseter diameter (HR, 0.12; 95% CI, 0.02–0.78) were associated with survival. Masseter diameter was the only factor associated with 90-day mortality after surgical resection (*P* = 0.044).

**CONCLUSIONS:**

GBM patients over the age of 79 have acceptable outcomes after resection, followed by adjuvant chemotherapy and RT. In addition to the treatment factors that predicted for survival, a decreased masseter diameter on preoperative imaging, a marker of sarcopenia, was associated with shorter overall survival and 90-day mortality after surgical resection.

## INTRODUCTION

The overall prognosis for patients with glioblastoma (GBM) can be improved with the use of surgical resection, chemotherapy, and radiotherapy (RT).^1^ However, concern has been raised that older patients with GBM might not respond as well to the standard treatment modalities, and elderly patients frequently do not meet the criteria for clinical trial enrollment.^2–4^ Older age has been a commonly reported poor prognostic factor.^5,6^ However, it is unclear whether older age has been considered a poor prognostic factor because of a poor response to therapy, aggressive tumor biology, patient frailty, or differences in patient management compared with younger cohorts. Compared with younger patients with GBM, elderly patients are less likely to undergo resection and less likely to receive postoperative adjuvant RT or chemotherapy, perhaps positioning these patients to have a poor prognosis compared with their younger counterparts.^7^ Moreover, as the general population has continued to age, the prevalence of older patients with GBM has continued to increase. Thus, the question regarding optimal treatment strategies has become increasingly relevant.

No universal definition for “elderly” has been determined, and studies have used different cutoffs to assign an “elderly” label to patients. Some studies have considering patients aged >65 years as “elderly,” although the median age for the diagnosis for GBM is 65 years.^8–15^ In addition, the age-adjusted incidence rates for GBM have been highest for the 75–84-year age group, and the average annual age-specific incidence rate is 9 per 100,000 persons for patients aged ≥85 years.^15^ At present, the average life expectancy is 78.7 years in the United States,^16^ and this threshold might, therefore, and this threshold may better serve to delineate patients who are at an increased risk of poor outcomes. The goal of the present study was to identify the treatment and patient factors, including imaging measures of sarcopenia, that would correlate with survival for patients older than the average age of life expectancy and to determine the effects of surgical intervention on a patient’s functional status. By identifying the preoperative factors within this elderly cohort associated with shorter survival outcomes, these data might help improve patient counseling and help identify higher surgical risk individuals.

## METHODS

### Patient Selection

The University of California, San Francisco, institutional review board approved the present study (approval no. 15–17500), and the University of California, San Francisco, tumor registry was searched for patients who met the following inclusion criteria: 1) age ≥79 years at surgery; 2) biopsy or resection performed of an intracranial IDH (isocitrate dehydrogenase) wild-type World Health Organization grade IV GBM at the initial diagnosis; and 3) no prior RT or chemotherapy before surgery. The search resulted in 110 patients who had undergone surgery between 1997 and 2020. An age cutoff of 79 years was used because of the average life expectancy of 78.7 years in the United States.^16^

### Patient and Tumor Variables

Patient, tumor, and outcomes data were collected retrospectively from the electronic medical records. The patient and tumor information for the cohort included age at surgery, sex, race/ethnicity, tumor laterality, location, O^6^-methylguanine-DNA methyltransferase (MGMT) methylation status, preoperative Karnofsky performance scale (KPS) score, preoperative laboratory values, and medical comorbidities. Tumor location was defined as the predominant lobe in which the tumor was located, unless the tumor spanned 2 cortical regions equally. Multifocal disease was defined by non-contiguous areas of enhancing disease. The MGMT status had been documented for 53 patients.

Of the 110 patients in the cohort, 102 had had imaging studies available for review to analyze the preoperative radiographic factors. The imaging features, including masseter diameter, temporalis diameter, sylvian fissure gap, and cortical mantle thickness, were retrospectively assessed from the preoperative magnetic resonance imaging (MRI) studies (Supplementary Figure 1). Masseter muscle diameter was assessed by measuring this musculature from medially to laterally, perpendicular to the mandible on axial T2-weighted MRI views at the level of the mandibular notch. The temporalis muscle diameter was assessed by measuring this musculature on axial contrast-enhanced T1-weighted MRI views perpendicular to the long axis of the temporal muscle at the level of the Sylvian fissure (anteroposterior landmark) and the orbital roof (craniocaudal landmark), in accordance with prior reports.^17^ Sylvian fissure gap was measured between the insula and overlying temporal operculum on axial T2-weighted sequences. The frontal cortical mantle thickness was measured from the edge of the frontal horn to the outer edge of the frontal lobe cortex. An average was taken of the bilateral measurements unless the tumor burden had distorted an imaging feature on 1 side. In these cases, only the measurement of the unaffected side was used. Of the 110 patients, 3 had had imaging studies that did not allow for an assessment of the masseter diameter, and 4 had had butterfly gliomas that prevented an accurate assessment of the frontal cortical mantle thickness. An analysis was performed to compare the blinded interobserver measurements of the masseter diameter with the measurements of the temporalis diameter, which had been previously reported as a measure of sarcopenia.^17,18^ Interobserver measurements of the masseter diameter correlated more strongly than did the temporalis diameter (*R*^*2*^, 0.786 vs. 0.63; Supplementary Figure 2).

### Treatment

The surgical approach and implementation of adjuvant therapy was determined by the patient or family wishes, tumor location and disease extent, and recommendations from the multidisciplinary team of treating physicians, which consisted of neurosurgeons, a neuro-oncologist, and a radiation oncologist. The patients were classified as having undergone biopsy for diagnosis (defined as any extent of resection <30%), subtotal total resection (STR) if >30% of tumor had been removed with persistence of residual enhancing tumor, or gross total resection (GTR) if all enhancing tumor had been removed. The extent of resection versus biopsy was determined by radiographic analysis by an attending neuroradiologist who was unaware of the outcomes, and all the patients had had postoperative MRI scans obtained within 48 hours after surgery. The patients in the cohort had either received no adjuvant therapy, adjuvant temozolomide (TMZ) alone, external beam RT alone, or a combination of adjuvant RT and concurrent TMZ therapy. RT was delivered either at a standard dose of 54–60 Gy or a reduced dose of <54 Gy. The decision regarding the RT dose was made by the treating neuro-oncologist and radiation oncologist. Of the 62 patients who had undergone postoperative RT, 50 had documentation of the radiation dose available.

### Outcomes

The primary outcomes of interest for the present study were overall survival (OS), postoperative KPS score, 90-day mortality, and 30-day complications from the date of surgery. Complications included medical and surgical complications and 30-day mortality. Reoperations were also recorded if they had occurred within 90 days after the initial surgery to treat a complication. The postoperative KPS score was documented at the first clinic follow-up after surgery.

### Statistical Analysis

Descriptive statistics were used to define the patient cohort, tumor characteristics, and clinical outcomes. A *t* test was used to compare the continuous patient age between the resection and biopsy subgroups. The Wilcoxon rank sum test was used to compare preoperative KPS scores between the resection and biopsy subgroups. The Pearson χ^2^ test was used to compare the nominal variables with an adequate sample size per group. The Fisher exact test was used to compare the tumor location between the resection and biopsy subgroups. A subgroup comparison of OS was performed using the Kaplan-Meier method, with the log-rank test used for the statistical comparisons. For masseter and temporalis diameters, the median value for the entire cohort was used to create a cutoff, and a comparison of survival between subgroups stratified by this cutoff was performed. Univariate Cox proportional hazard analyses were first performed to identify the factors associated with OS. All variables meeting a threshold *P* value of < 0.1 on univariate analysis were included in the multivariate Cox model. Variables meeting a threshold *P* value of < 0.05 in the multivariate Cox proportional hazards model were considered significantly associated with survival. Univariate and multivariate nominal logistic regression analyses were performed to identify the factors associated with 30-day complications after surgery. A univariate nominal logistic regression analysis was performed to identify the factors associated with 90-day mortality after resection. Only 1 variable (masseter diameter) was significantly associated with 90-day mortality; thus, multivariate analysis was not performed. A *P* value of < 0.05 was considered statistically significant for all analyses. Analysis was performed using JMP Pro, version 15 (SAS Institute, Cary, North Carolina, USA).

## RESULTS

### Patient Demographics and Tumor Characteristics

Cohort details are listed in Table 1. The cohort included 65 males (59.1%), and the mean age at surgery was 82.8 years (range, 79–94.1 years). The median preoperative KPS score was 80 (range, 40–90), and 15%, 29%, and 6% of the cohort had had diabetes mellitus, cardiac disease, and pulmonary disease, respectively. Overall, 95.5% of the tumors were unilateral (left, 49.1%; right, 46.4%), and 4.5% of the tumors involved both hemispheres. The most common tumor location was the frontal lobe (32.7%), and 16 patients (14.5%) had multifocal disease, consisting of noncontiguous regions of contrast enhancement on imaging studies. Of the 53 patients with MGMT methylation status available, 73.6% and 26.4% had had methylated and nonmethylated MGMT promoters, respectively.

### Treatment Details and Surgical Outcomes

Of the 110 patients in the cohort, 37 (33.6%) had undergone biopsy and 73 (66.4%) had undergone resection (Table 1). Clinical details were available during follow-up for 91 patients. Of the 91 patients with documented follow-up, 66 (72.5%) had received adjuvant therapy (TMZ and/or RT). Of the 66 patients, 26 (28.5%) had received either TMZ or RT (22 had received RT only and 4 had received TMZ only) and 40 (44%) had received both TMZ and RT. For the 62 patients who had undergone RT, detailed dosing information was available for 50 patients. A standard dose of 54–60 Gy was used for 38% of the 50 patients, and a hypofractionated regimen of <54 Gy was used for 62% of the patients.

Of the 110 patients, 26 (23.6%) had a medical or surgical complication, or 30-day mortality. The complications included subdural hematoma (*n* = 1), intraparenchymal or intraventricular hemorrhage (*n* = 3), worsening hydrocephalus requiring shunting (*n* = 1), worsening cerebral edema requiring readmission (*n* = 1), pulmonary embolism (*n* = 3), lower extremity deep venous thrombosis (*n* = 1), seizures leading to prolonged hospitalization or readmission (*n* = 5), urinary tract infection (*n* = 5), pneumonia (*n* = 3), acute kidney injury (*n* = 1), delirium prolonging hospitalization (*n* = 2), and sepsis (*n* = 1). Six patients (5.5% of cohort) died within 30 days after surgery. One patient (0.9%) had required operative intervention within 90 days postoperatively, which involved ventriculoperitoneal shunt placement for worsening hydrocephalus.

By the last follow-up, 94 patients (85.5% of the cohort) had died. The median survival after surgery was 4.9 months (95% confidence interval [CI], 3.9–7.4). The estimated 3-, 6-, and 12-month survival rates were 69.9%, 47.3%, and 24.8%, respectively.

### Comparison of Biopsy and Resection Groups

**Figure 1.**
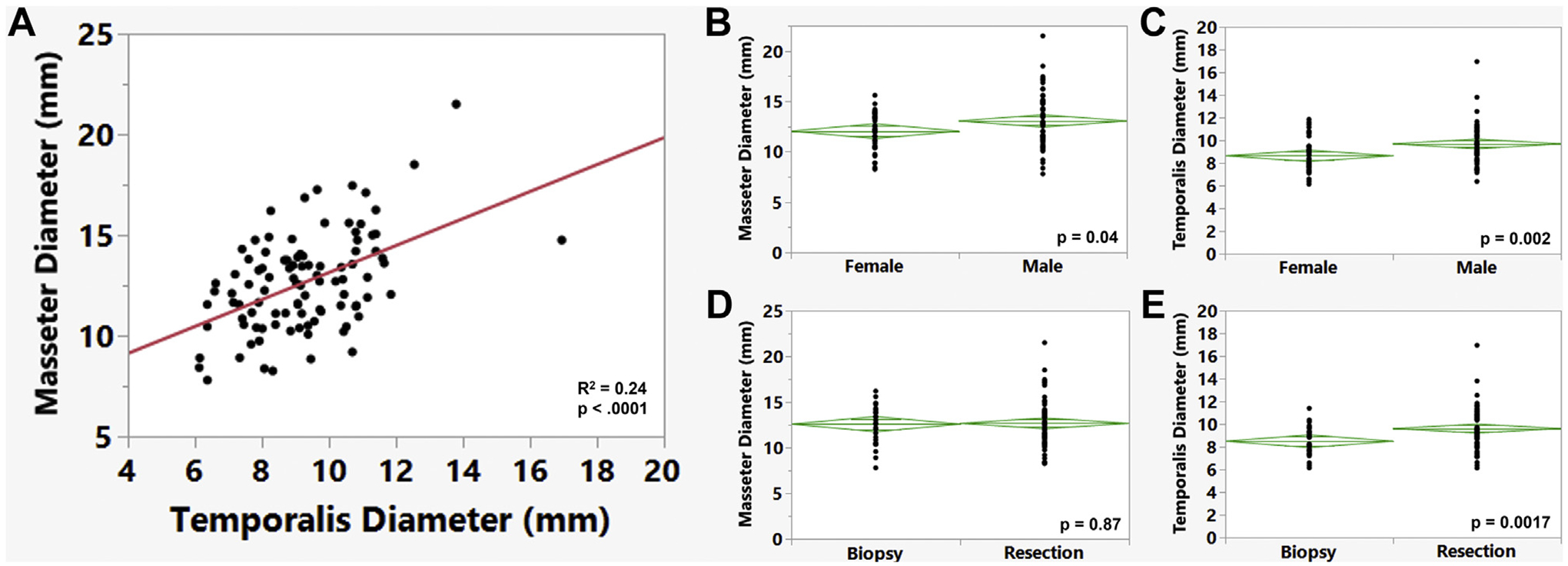
Correlation between temporalis diameter and masseter diameter and other patient factors. (**A**) Moderate correlation was found between these 2 markers of muscle mass (*P* < 0.0001). (**B,C**) The masseter and temporalis diameter were both associated with sex. (**D,E**) Although temporalis diameter was associated with surgical intervention selection (*P* = 0.0017), the masseter diameter was not (*P* = 0.87).

Patients who had undergone biopsy versus resection were compared to determine the patient, tumor, and outcome differences (Table 1). The mean age, preoperative KPS score, MGMT status, race/ethnicity, preoperative laboratory values, and prevalence of medical comorbidities did not differ significantly between the 2 groups. The biopsy group had significantly more women than did the resection group (54.1% vs. 34.3%; *P* = 0.046). The tumor location also varied between the approaches. The biopsy group had 5 patients with bilateral disease and 2 patients with tumors within the basal ganglia not amenable to surgical resection. Five patients who had multifocal disease within a single cerebral hemisphere had undergone surgical resection because of a dominant enhancing mass with a significant mass effect, which was the target of surgery. The use of adjuvant therapy and the use of reduced dose RT did not differ between groups. Of the preoperative imaging features, only a decreased temporalis diameter was significantly associated with biopsy (Figure 1; *P* = 0.0017). The masseter diameter, cortical mantle thickness, and Sylvian fissure gap were not associated with the type of surgical intervention. However, the temporalis thickness differed by patient sex (Figure 1), and women, who had smaller temporalis diameter, were more likely to have undergone biopsy (Table 1). On multivariate analysis incorporating the patient and treatment factors that met a threshold of *P* < 0.1 on univariate analysis, only a larger temporalis diameter was associated with resection instead of biopsy (unit odds ratio [OR], 2.02; 95% CI, 1.00–4.10; *P* = 0.03).

**Figure 2.**
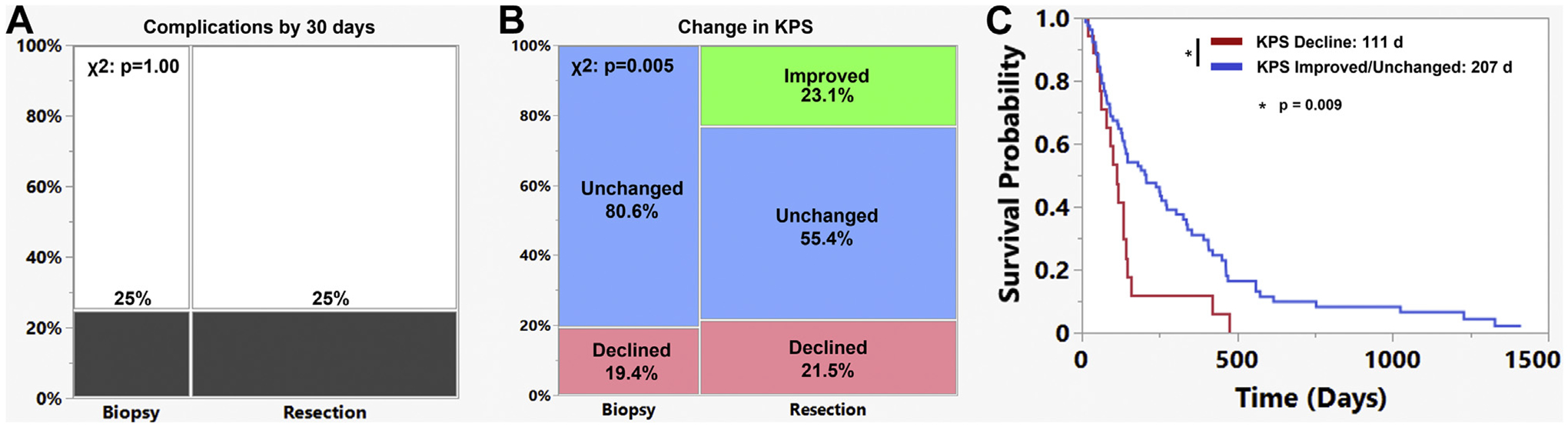
Effects of resection and biopsy on complications and Karnofsky performance scale (KPS). (**A**) The incidence of 30-day complications was 25% and 25% for the biopsy and resection groups, respectively (*P* = 1.00). (**B**) Of the patients who had undergone biopsy, 19.4% had a decline in the postoperative KPS score by the first follow-up appointment. For the patients who had undergone undergoing resection, 23.1% had had improvement in the KPS score and 21.5% had a decline in the KPS score by the first follow-up. (**C**) The patients with a decline in the postoperative KPS score had worse overall survival (*P* = 0.009).

There were no differences in the rate of 30-day complications between the biopsy and resection groups (25% vs. 25%; *P* = 1.00; Table 1 and Figure 2A). For patients who had undergone biopsy, 19.4% and 80.6% had had a decline or no change in the KPS score, respectively. Of the patients who had undergone resection, 21.5%, 55.4%, and 23.1% had a decline, no change, or improvement in KPS score, respectively (Figure 2B; χ^2^, *P* = 0.005). Thus, 78.5% of the resection cohort had experienced an improvement or no change in their postoperative KPS score by first follow-up. In terms of discharge location, 53.6% of the patients who had undergone resection and 55.9% of the patients who had undergone biopsy were discharged home (*P* = 0.83). The hospital length of stay also did not differ between the resection and biopsy groups (7.6 vs. 5.6 days; *P* = 0.11). The resection group had a significantly longer median OS than patients in the biopsy group (resection vs. biopsy, 7.4 vs. 3.0 months; *P* = 0.001).

### Patient and Treatment Factors Associated with OS

The effect of surgical resection and adjuvant therapy on OS was evaluated first (Supplementary Figure 3). Both GTR and STR were associated with prolonged OS compared with biopsy only (GTR vs biopsy, 13.1 vs. 3.0 months; log-rank *P* = 0.0012; STR vs. biopsy, 6.0 vs. 3.0 months; log-rank *P* = 0.017; Supplementary Figure 3A). However, no statistically significant difference was found in survival between the GTR and STR subgroups (13.1 vs. 6.0 months; *P* = 0.18). Additionally, both combination adjuvant therapy with RT and TMZ and monotherapy with either RT or TMZ alone were associated with prolonged survival compared with no postoperative therapy (RT plus TMZ vs. no therapy, 13.1 vs. 2.0 months; log-rank *P* < 0.0001; RT or TMZ vs. no therapy, 6.9 vs. 2.0 months; log-rank *P* = 0.0003; Supplementary Figure 3B). The use of RT plus TMZ was associated with longer survival than the use of RT or TMZ monotherapy (*P* = 0.0003). When considering both the surgical approach and adjuvant therapy, resection with adjuvant therapy led to longer survival than did biopsy with adjuvant therapy (11.2 vs. 4.9 months; log-rank *P* = 0.0041). However, no difference in survival was found between biopsy and resection if patients did not received additional postoperative adjuvant therapy (Supplementary Figure 3C).

Patients who had a stable or an improved KPS score postoperatively had significantly longer survival than that of the patients with a decline in functional status after either biopsy or resection (improved or unchanged KPS score vs. decreased KPS score, 6.9 vs. 3.7 months; *P* = 0.009; Figure 2C). This remained significant whether considering the subgroup of patients who had undergone resection (*P* = 0.003) or biopsy (*P* = 0.03). However, patients who had experienced a decline in the KPS score postoperatively were also less likely to receive adjuvant RT or chemotherapy (improved or unchanged KPS vs. decreased KPS, 77.6% vs 50%; *P* = 0.02). Patients who were discharged home after surgery also had longer survival compared with that of the patients discharged to a rehabilitation facility or skilled nursing facility or transferred back to their referring hospital (home vs. rehabilitation, skilled nursing facility, outside hospital, 8.3 vs. 4.3 months; *P* = 0.01; Supplementary Figure 3D).

**Figure 3.**
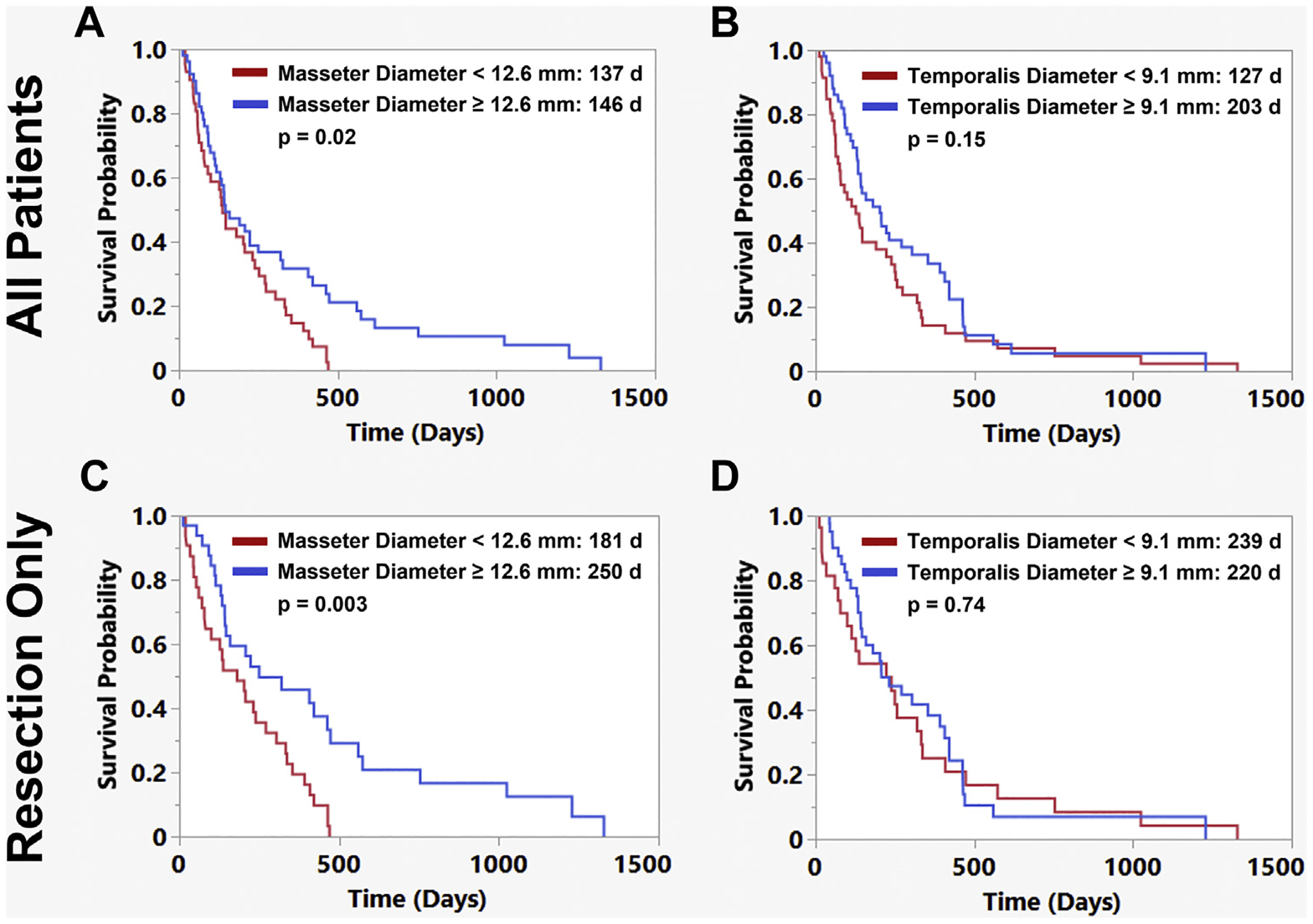
Comparison of masseter and temporalis diameter correlation with outcomes and patient and treatment factors. (**A,B**) When using the median value of the masseter or temporalis diameter as a cutoff (12. 6 mm and 9.1 mm, respectively), a masseter diameter of <12.6 mm was associated with worse overall survival (*P* = 0.02) and a temporalis diameter of <9.1 mm was not (*P* = 0.15) for the entire cohort. (**C,D**) When examining the subset of patient who had undergone resection, a masseter diameter of <12.6 mm was still associated with worse survival (*P* = 0.003) and the temporalis diameter was not (*P* = 0.74).

The masseter diameter was also significantly associated with OS in the entire cohort and in the resection subgroup (Figure 3A and C). Using the median value of the masseter diameter as a cutoff, patients with a masseter diameter <12.6 mm had significantly shorter OS than patients with a masseter diameter of ≥12.6 mm (*P* = 0.023; Figure 3C). The masseter diameter correlated only weakly with patient age and preoperative KPS score (Supplementary Figure 4). The temporalis thickness was not associated with survival for either the entire cohort or within the resection subgroup (Figure 3B and D). Cortical mantle thickness and Sylvian fissure gap were also not associated with OS.

A univariate Cox proportional hazard analysis was performed to identify the patient, tumor, and treatment factors associated with OS (Table 2). On univariate analysis, age (hazard ratio [HR], 8.13), preoperative KPS score (HR, 0.19), postoperative KPS score (HR, 0.15), adjuvant therapy (RT or TMZ vs. none: HR, 0.18; RT plus TMZ vs. none: HR, 0.06), masseter diameter (HR, 0.21), preoperative hemoglobin (HR, 0.39), and surgical resection (HR, 0.49) were associated with OS. On multivariate analysis, age (HR, 7.97; 95% CI, 1.63–36.3), multifocality (HR, 2.7; 95% CI, 1.14–6.40), adjuvant therapy (RT or TMZ vs. none: HR, 0.12; 95% CI, 0.05–0.3; RT plus TMZ vs. none: HR, 0.05; 95% CI, 0.02–0.14), surgical resection (HR, 0.46; 95% CI, 0.24–0.9), and masseter diameter (HR, 0.12; 95% CI, 0.02–0.78) were associated with OS. When applying this model to the resection subgroup, the masseter diameter was still significantly associated with OS (HR, 0.13; 95% CI, 0.02–0.83; *P* = 0.034; Supplementary Table 1).

### Factors Associated with 30-Day Complications and 90-Day Mortality

Univariate nominal logistic regression analysis was performed to identify the factors associated with the 30-day postoperative complications (Table 3). On univariate analysis, female sex (OR, 3.02) and preoperative KPS score (OR, 0.11) were associated with 30-day complications. On multivariate analysis, female sex (OR, 3.10; 95% CI, 1.13–8.50) and a lower preoperative KPS score (OR, 0.11; 95% CI, 0.02–0.70) were associated with an increased risk of complications.

Because practitioners often choose to avoid aggressive surgical intervention for patients without a life expectancy >3 months, which corresponds to the recovery time after a craniotomy, univariate analysis was conducted to determine the preoperative clinical and imaging correlates of 90-day mortality after resection (Table 4). The masseter diameter was the only significant correlate of 90-day mortality (*P* = 0.044). The masseter diameter was also associated with 60-day mortality (*P* = 0.044). Older age (*P* = 0.066) and a lower preoperative KPS score (*P* = 0.059) both demonstrated a trend toward statistical significance for an association with 90-day mortality.

## DISCUSSION

The goals of the present study were to identify the predictors of survival for patients with a diagnosis of IDH wild-type GBM whose age was older than the average life expectancy (78.7 years), examine the effects of surgical intervention on a patient’s functional status, and evaluate whether preoperative imaging markers of sarcopenia and brain atrophy were predictors of OS and postoperative complications. In the present cohort of 110 patients, any adjuvant therapy and undergoing resection were both associated with improved OS. When accounting for both forms of treatment, the patients who had undergone resection followed by adjuvant therapy survived longer than those who had undergone biopsy followed by adjuvant therapy, with more long-term survivors at 2 years (estimated 2-year survival, 15.7% vs. 0%). However, no differences were found in survival between the resection and biopsy groups for patients who had not undergone adjuvant therapy. Although the rates of a KPS score decline were similar between the biopsy and resection groups, >20% of patients had an improvement in the KPS score by the first clinical follow-up after resection. Furthermore, the masseter diameter, a marker of sarcopenia, was associated with OS in this elderly cohort of patients with GBM and was the only factor that was significantly associated with mortality within 90 days of surgical resection. The temporalis diameter was associated with higher rates of biopsy on multivariate analysis, suggesting the diameter had influenced the treatment decisions. However, this preoperative imaging feature was not associated with the survival outcomes in this elderly cohort. It is, therefore, possible that the temporalis muscle thickness, which is visible, serves as a subjective clinical measure of fitness when meeting with patients and, thus, affects surgical decision-making. However, in the present cohort of elderly patients, the masseter diameter, which is less distinct on bedside evaluation, was the more relevant imaging feature for sarcopenia and correlated with survival.

### Comparison with Reported Studies

The overall prognosis for elderly patients with GBM has been thought to be worse than that for their younger counterparts.^5,6,19^ For patients who had undergone resection plus adjuvant therapy in the present cohort, the median survival was 337 days. Although this was a shorter duration compared with historical groups of younger patients with GBM, it was still 4–5 times longer than that for the patients who had undergone biopsy alone and almost double that for the patients who had undergone biopsy before receiving adjuvant therapy. However, despite the benefits of surgical resection, 33.6% of the patients had undergone biopsy only and 27.5% had undergone no postoperative adjuvant therapy, both of which are more frequent management paradigms for the elderly GBM population.^20^

Despite a general concern that elderly patients might not tolerate a more aggressive surgical procedure, prior studies have suggested improvements in survival after aggressive surgical resection.^11,19,21^ A study by Lopez-Rivera et al.^22^ using the Surveillance, Epidemiology, and End Results 18 registry to identify patients aged ≥65 years with GBM found that both GTR and supratotal resection were associated with improved survival even for octogenarian patients.^22^ Additionally, prior systematic analyses have demonstrated improved survival for elderly patients who had undergone chemotherapy plus RT.^13^

In the present study, we focused on patients who had lived longer than the average life expectancy because this population still comprises a significant portion of adult patients with GBM. The age-adjusted incidence rates of GBM are highest for the 75–84-year age group. For patients aged ≥85 years, the average annual age-specific incidence rate has remained 9 per 100,000 persons.^15^ Thus, the question for the treatment of elderly patients is common and affects a significant proportion of all patients with GBM. Despite using a stricter definition of elderly, in accordance with the average life expectancy, we demonstrated a benefit for resection compared with biopsy when patients were able to undergo postoperative adjuvant therapy. There is also a potential concern that resection may precipitate a decline in functional status in elderly patients with GBM. However, overall, we found no difference in the rates of KPS score decline between biopsy and resection, and some patients had actually experienced an improvement in the KPS score with resection.

For any surgical procedure, risk assessment and patient selection are critical to optimizing the treatment plans and patient outcomes. Traditionally, patient age has been viewed as an important variable for predicting adverse events, and surgical decision-making has been influenced by the surgeon’s view of the patient’s comorbidities and disease burden as well as the patient’s treatment goals. The determination of patient frailty is a valuable tool and has been shown to be more accurate than the chronological age for predicting postoperative complications, in-hospital mortality, and length of stay.^23^ No universal metric has been approved for assessing frailty; however, numerous survey metrics (e.g., 11-item modified frailty index) and comorbidity indexes (e.g., Charlson comorbidity index) exist. The clinical use of these metrics in the neuro-oncology realm has remained underused despite prior reports demonstrating the association of brain tumor patient frailty with the outcomes, including discharge disposition, length of stay, and mortality.^24–26^

Sarcopenia has been cited as a negative prognosticator for patients with GBM and in other oncologic contexts and could serve as a surrogate marker for frailty.^17,18,27^ Numerous reasons exist for why patients with GBM might have sarcopenia, including steroid use, poor nutritional intake, and decreased mobility owing to neurologic deficits, all of which can lead to muscle atrophy. Postoperatively, patients may develop loss of muscle mass related to systemic chemotherapy or focal fibrosis of the temporalis muscle if it is within the radiation field. In the present cohort, a decreased masseter diameter was associated with worse OS and 90-day mortality. This imaging feature can be evaluated on preoperative MRI scans and can be easily incorporated into treatment decision making. A decreased temporalis thickness was associated with increased rates of biopsy, suggesting that this visible feature was associated with a less aggressive treatment choice by surgeons. However, unlike the masseter diameter, the temporalis thickness did not correlate with the survival outcomes, and neurosurgeons should be cautious in making surgical decisions using temporalis thickness as a frailty marker. The findings from prior studies have been mixed regarding whether temporalis thickness is associated with survival after GBM treatment, with some studies reporting associations with survival only for patients with progressive or secondary GBM.^17,18,28–32^ Prior studies in other oncologic contexts have demonstrated that different measures of sarcopenia often do not strongly correlate and have different abilities to predict outcomes of interest.^33–35^

Neurosurgeons and neuro-oncologists are required to make complex treatment decisions for elderly GBM patients while taking into account the patient’s goals of care. Unfortunately, the data from prior clinical trials is often insufficient to apply to this older cohort of patients. These data highlight the importance of adjuvant therapy following surgical resection and the benefit of surgical resection compared to a biopsy alone. Complication rates, although higher than those seen in younger patients, are typically medical in nature and should be expected when designing adjuvant treatment plans for these older patient populations. Preoperative frailty markers, such as sarcopenia, may help guide those discussions, and based on findings from this report, patients with significant sarcopenia based on decreased masseter diameter may not be appropriate surgical resection candidates.

### Study Limitations

The present study had several inherent limitations. First, given its retrospective nature, observation and selection bias could have been present in our cohort. We tried to limit the selection bias by including all the patients who had been treated at the center during the study period. However, treatment modalities used in the cohort were determined from the multidisciplinary team recommendations and patient wishes and, therefore, could not be standardized across the cohort. Second, the present study was a single-center study. Thus, the treatment algorithms, criteria for selecting patients for surgery, and surgical outcomes might differ from those from other centers. Third, although serum markers of sarcopenia were of interest, we were unable to comprehensively assess these because many sarcopenia-related laboratory tests were not a part of routine clinical care. Finally, fewer than one half of the patients had the MGMT status available, limiting this component of the analysis.

## CONCLUSIONS

Despite a general concern that elderly patient may not tolerate more aggressive treatment interventions, GBM patients diagnosed past the average age of life expectancy still fare better when undergoing resection followed by adjuvant chemotherapy and radiation therapy. Resection can be performed with no added risk of decline in KPS postoperatively when compared to biopsy. In addition to treatment factors that predict survival, decreased masseter diameter, a marker of sarcopenia, is associated with shorter overall survival and 90-day mortality after performing a surgical resection. This imaging feature can be evaluated on preoperative MRI scans and can be easily incorporated into treatment decision making.

## Supplementary Material

1

## Figures and Tables

**Table 1.** Cohort Details and Comparison of Patients Undergoing Biopsy Versus Resection

Variable	All Patients (*n* = 110)	Biopsy (*n* = 37)	Resection (*n* = 73)	*P* Value
Age* (years)	82.8 (79–94.1)	82.8 (79.6–91.5)	82.8 (79–94.1)	0.96
Sex				0.046
Male	65 (59.1)	17 (45.9)	48 (65.7)	
Female	45 (40.9)	20 (54.1)	25 (34.3)	
Laterality				0.002
Left	54 (49.1)	20 (54.1)	34 (46.6)	
Right	51 (46.4)	12 (32.4)	39 (53.4)	
Bilateral	5 (4.5)	5 (13.5)	0 (0)	
Location				0.0045
Frontal	36 (32.7)	11 (29.7)	25 (34.3)	
Parietal	9 (8.2)	2 (5.4)	7 (9.6)	
Temporal	16 (14.5)	2 (5.4)	14 (19.2)	
Occipital	4 (4.6)	0 (0)	4 (5.5)	
Insula	1 (0.9)	1 (2.7)	0 (0)	
Cerebellum	2 (1.8)	0 (0)	2 (2.7)	
Basal ganglia	2 (1.8)	2 (5.4)	0 (0)	
>1 Lobe	24 (21.8)	8 (21.6)	16 (21.9)	
Multifocal	16 (14.5)	11 (29.7)	5 (6.9)	
MGMT status†				0.06
Methylated	39 (73.6)	9 (56.3)	30 (81.1)	
Nonmethylated	14 (26.4)	7 (43.7)	7 (18.9)	
Mean preoperative KPS score‡	73.0	69.2	75.0	0.09
Preoperative KPS score group				
90	19 (18.3)	7 (19.4)	12 (17.8)	
80	38 (36.5)	9 (25)	29 (42.7)	
70	21 (20.2)	5 (13.9)	16 (23.5)	
60	12 (11.5)	6 (16.7)	6 (8.8)	
50	9 (8.7)	7 (19.4)	2 (2.9)	
40	5 (4.8)	2 (5.6)	3 (4.4)	
Race/ethnicity§				0.18
White/Caucasian	79 (75.2)	28 (82.4)	51 (71.8)	
Black/African American	7 (6.7)	3 (8.8)	4 (5.6)	
Hispanic/Latino	5 (4.8)	2 (5.9)	3 (4.2)	
Asian/Pacific Islander	14 (13.3)	1 (2.9)	13 (18.3)	
CCI*	6.5 (5–9)	6.5 (5–8)	6.5 (5–9)	0.96
BMI* (kg/m^2^)	26.1 (17.8–37.9)	26.2 (21.5–32.8)	26.1 (17.8–37.9)	0.89
Diabetes‖	15 (15)	6 (18.2)	9 (13.4)	0.53
Cardiac disease‖	29 (29)	10 (30.3)	19 (28.4)	0.84
Pulmonary disease‖	6 (6)	3 (9.1)	3 (4.5)	0.36
Preoperative hemoglobin*	13.1 (9.8–16.6)	12.8 (10.6–15.1)	13.3 (9.8–16.6)	0.12
Preoperative WBC count*	10.5 (3.7–24.9)	10.2 (3.7–24.9)	10.6 (3.9–23.8)	0.70
Preoperative serum creatinine*	0.93 (0.49–2.32)	0.96 (0.49–1.73)	0.91 (0.51–2.32)	0.56
Masseter diameter¶ (mm)	12.6 (7.8–21.5)	13 (7.8 –16.3)	12.3 (8.3–21.5)	0.87
Temporalis diameter¶ (mm)	9.1 (6.1–16.9)	8.2 (6.1–11.4)	9.4 (6.1–16.7)	0.0017
Sylvian fissure gap¶ (mm)	5.9 (3.4–12.3)	5.5 (3.7–10.0)	6.1 (3.4–12.3)	0.35
Cortical mantle thickness¶ (mm)	32.2 (25.4–38.3)	32.1 (27.5–38.3)	32.4 (25.4–36.7)	0.95
Adjuvant therapy#				0.42
None	25 (27.5)	10 (35.7)	15 (23.8)	
TMZ or RT	26 (28.5)	6 (21.4)	20 (31.8)	
RT and TMZ	40 (44)	12 (42.9)	28 (44.4)	
Radiation dose**				0.57
Standard dose (54–60 Gy)	19 (38)	7 (43.8)	12 (35.3)	
Reduced dose (<54 Gy)	31 (62)	9 (56.3)	22 (64.7)	
30-Day complications††	26 (25)	8 (25)	18 (25)	1.00
Death by last follow-up	94 (85.5)	31 (83.8)	63 (86.3)	0.72
Median OS‡‡ (months)	4.9 (3.9–7.4)	3.0 (2.1–4.7)	7.4 (4.6–10.7)	0.001
Discharge home	56 (54.4)	19 (55.9)	37 (53.6)	0.83
Hospital length of stay* (days)	6.9 (1–42)	5.6 (1–16)	7.6 (2–42)	0.11

Data presented as *n* (%), unless noted otherwise.

MGMT, O^6^-methylguanine-DNA methyltransferase; KPS, Karnofsky performance scale; CCI, Charlson comorbidity index; BMI, body mass index; WBC, white blood cell; TMZ, temozolomide; RT, radiotherapy; OS, overall survival.

* Data presented as mean (range).

† MGMT status known for 53 patients.

‡ Documentation missing for 6 patients.

§ Documentation of race/ethnicity missing for 5 patients.

‖ Documentation missing for 10 patients.

¶ Data presented as median (range).

# Documentation missing for 19 patients lost to follow-up.

** Of the 62 patients who had received postoperative RT, 50 had had documentation available for the radiation dose.

†† Documentation missing for 12 patients lost to follow-up.

‡‡ Data presented as median (95% confidence interval).

**Table 2.** Univariate and Multivariate Cox Proportional Hazard Analysis for Predictors of Survival

	Univariate Analysis		Multivariate Analysis	
Variable	HR (95% CI)	*P* Value	HR (95% CI)	*P* Value
Age	8.13 (2.55–24.57)	0.0003	7.97 (1.63–36.3)	0.0083
Sex (female vs male)	0.89 (0.59–1.35)	0.58	–	–
Laterality (vs. bilateral)			–	–
Left	0.92 (0.33–2.57)	0.87		
Right	1.15 (0.41–3.24)	0.79		
Multifocal	1.81 (0.98–3.35)	0.06	2.70 (1.14–6.40)	0.024
Minority (vs. Caucasian)	0.50 (0.28–0.87)	0.015	0.79 (0.39–1.60)	0.52
MGMT methylated (vs. nonmethylated)	0.84 (0.41–1.70)	0.62	–	–
Preoperative KPS score	0.19 (0.09–0.43)	<0.0001	0.48 (0.13–1.83)	0.27
Postoperative KPS score	0.15 (0.07–0.34)	<0.0001	1.23 (0.28–5.50)	0.78
Masseter diameter	0.21 (0.05–0.88)	0.041	0.12 (0.02–0.78)	0.033
Temporalis diameter	0.91 (0.79–1.03)	0.14	–	–
Sylvian fissure gap	1.09 (0.43–2.63)	0.85	–	–
Cortical mantle thickness	0.42 (0.12–1.42)	0.16	–	–
CCI	2.68 (0.77–8.52)	0.11	–	–
Preoperative WBC count	0.90 (0.25–3.01)	0.87	–	–
Preoperative hemoglobin	0.39 (0.14–1.06)	0.069	0.53 (0.12–2.33)	0.40
Preoperative serum creatinine	2.07 (0.38–10.02)	0.38	–	–
BMI	1.31 (0.32–5.00)	0.70	–	–
Diabetes	0.78 (0.42–1.46)	0.43	–	–
Cardiac disease	0.96 (0.59–1.56)	0.86	–	–
Pulmonary disease	1.30 (0.53–3.24)	0.58	–	–
Adjuvant therapy (vs. none)				<0.0001
RT or TMZ	0.18 (0.09–0.36)	<0.0001	0.12 (0.05–0.30)	
RT plus TMZ	0.06 (0.03–0.13)	<0.0001	0.05 (0.02–0.14)	
Reduced RT dose (vs. standard)	1.08 (0.58–2.02)	0.81	–	–
Resection (vs. biopsy)	0.49 (0.32–0.77)	0.003	0.46 (0.24–0.90)	0.023
Complication ≤30 days	1.21 (0.71–2.06)	0.49	–	–

HR, hazard ratio; CI, confidence interval; MGMT, O^6^-methylguanine-DNA methyltransferase; KPS, Karnofsky performance scale; CCI, Charlson comorbidity index; WBC, white blood cell; BMI, body mass index; RT, radiotherapy; TMZ: temozolomide.

**Table 3.** Univariate and Multivariate Analysis of Predictors of 30-Day Complications

	Univariate Analysis		Multivariate Analysis	
Variable	OR (95% CI)	*P* Value	OR (95% CI)	*P* Value
Age	3.39 (0.39–29.52)	0.27	–	–
Sex (female vs. male)	3.02 (1.21–7.56)	0.02	3.10 (1.13–8.50)	0.03
Laterality (vs. bilateral)			–	–
Left	0.87 (0.08–9.20)	0.91		
Right	1.14 (0.11–11.85)	0.92		
Minority (vs. Caucasian)	1.46 (0.54–3.96)	0.45	–	–
MGMT methylated (vs. nonmethylated)	3.25 (0.63–16.79)	0.13	–	–
Masseter diameter	0.21 (0.01–3.90)	0.28	–	–
Temporalis diameter	0.82 (0.60–1.10)	0.17	–	–
Sylvian fissure gap	3.17 (0.41–24.37)	0.27	–	–
Cortical mantle thickness	0.68 (0.07–6.91)	0.74	–	–
Preoperative KPS score	0.011 (0.02–0.65)	0.013	0.11 (0.02–0.70)	0.02
CCI	0.80 (0.08–8.31)	0.85	–	–
BMI	0.12 (0.01–2.11)	0.13	–	–
Diabetes	1.75 (0.53–5.83)	0.37	–	–
Cardiac disease	0.36 (0.11–1.16)	0.09	0.37 (0.11–1.28)	0.12
Pulmonary disease	1.48 (0.25–8.61)	0.67	–	–
Resection (vs. biopsy)	1 (0.38–2.62)	1.00	–	–

OR, odds ratio; CI, confidence interval; MGMT, O^6^-methylguanine-DNA methyltransferase; KPS, Karnofsky performance scale; CCI, Charlson comorbidity index; BMI, body mass index.

**Table 4.** Preoperative Factors Associated with Death ≤90 Days After Resection

Variable	≤90 Days (*n* = 9)	>90 Days (*n* = 28)	*P* Value
Mean age (years)	84.1 ± 0.7	82.5 ± 0.4	0.066
Sex			0.33
Male	12 (75)	34 (61.8)	
Female	4 (25)	21 (38.2)	
Laterality			0.17
Left	5 (31.2)	28 (50.9)	
Right	11 (68.8)	27 (49.1)	
Multifocal disease	1 (6.3)	4 (7.3)	0.89
Masseter diameter	11.6 ± 0.7	13.1 ± 0.4	0.044
Temporalis diameter	9.0 ± 0.3	9.5 ± 0.2	0.20
Sylvian fissure gap	7.2 ± 0.5	6.4 ± 0.3	0.20
Cortical mantle thickness	32.3 ± 0.7	32.3 ± 0.4	0.94
Preoperative KPS score*	69.3 ± 0.3	76.3 ± 0.2	0.059
Preoperative KPS score group			
90	1 (7.1)	11 (21.2)	
80	7 (50)	20 (38.5)	
70	2 (14.3)	14 (26.9)	
60	1 (7.1)	5 (9.6)	
50	0 (0)	2 (3.8)	
40	3 (21.5)	0 (0)	
Race/ethnicity†			0.16
Caucasian/white	13 (86.7)	37 (68.5)	
Minority	2 (13.3)	17 (31.5)	
CCI	6.4 ± 0.1	6.5 ± 0.1	0.80
BMI	26.1 ± 1.5	26.0 ± 0.8	0.99
Diabetes‡			0.09
Yes	0 (0)	9 (17.7)	
No	14 (100)	42 (82.3)	
Cardiac disease‡			0.25
Yes	2 (14.3)	15 (29.4)	
No	12 (85.7)	36 (70.6)	
Pulmonary disease‡			0.61
Yes	1 (7.1)	2 (3.9)	
No	13 (92.9)	49 (96.1)	

Data presented as mean ± standard error or *n* (%).

KPS, Karnofsky performance scale; CCI, Charlson comorbidity index; BMI, body mass index.

* Documentation missing for 6 patients.

† Race/ethnicity documentation missing for 5 patients.

‡ Documentation missing for 10 patients.

## Abbreviations and Acronyms

CI: Confidence interval
GBM: Glioblastoma
GTR: Gross total resection
HR: Hazard ratio
KPS: Karnofsky performance scale
MGMT: O^6^-methylguanine-DNA methyltransferase
MRI: Magnetic resonance imaging
OR: Odds ratio
OS: Overall survival
RT: Radiotherapy
STR: Subtotal resection
TMZ: Temozolomide
