# Supplementary material for: Sarcopenia Diagnosed Using Masseter Muscle Diameter as a Survival Correlate in Elderly Patients with Glioblastoma
Source: World Neurosurg. Author manuscript; Available in PMC 2022 Jul 15. (PMC9284942; doi:10.1016/j.wneu.2022.02.038)
Supplement: 1 [file NIHMS1822829-supplement-1.pdf]

SUPPLEMENTARY DATA

Supplemental Table 1. Multivariate Cox Proportional Hazard Analysis for Predictors of Survival for Resection Subgroup

	Multivariate Analysis	
Variable	HR (95% CI)	*P* Value
Age	9.85 (1.27—71.05)	0.025
Multifocal	4.63 (1.38—15.57)	0.013
Minority (vs. Caucasian)	0.78 (0.36—1.73)	0.55
Preoperative KPS score	1.06 (0.21—6.10)	0.95
Postoperative KPS score	2.29 (0.56—9.76)	0.25
Masseter diameter	0.13 (0.02—0.83)	0.034
Adjuvant therapy (vs. none)		<0.0001
RT or TMZ	0.07 (0.02—0.22)	
RT and TMZ	0.02 (0.004—0.08)	
Preoperative hemoglobin	0.63 (0.13—3.06)	0.57

HR, hazard ratio; CI, confidence interval; KPS, Karnofsky performance scale; RT, radiotherapy; TMZ: temozolomide.

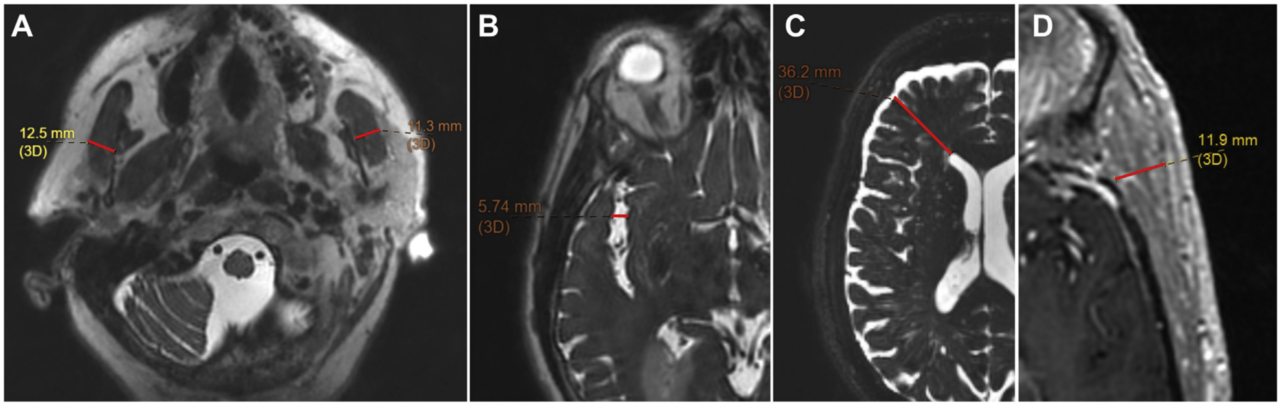

Supplementary Figure 1. Measurements of masseter diameter at the mandibular notch (**A**), sylvian fissure gap (**B**), cortical mantle thickness (**C**), and temporalis thickness (**D**).

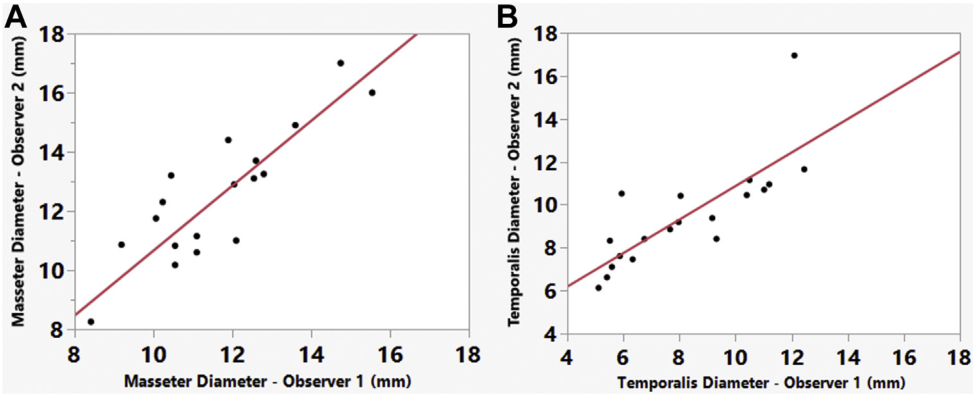

Supplementary Figure 2. Interobserver measurements of masseter diameter correlated more strongly than did measurements of the temporalis diameter. (**A**) Masseter diameter measurements closely correlated between observers who were unaware of the other clinical features of the cohort ($R^2 = 0.786$; $P < 0.0001$). (**B**) Temporalis diameter measurements also closely correlated between observers but not to the same degree as did the masseter diameter ($R^2 = 0.63$; $P < 0.0001$).

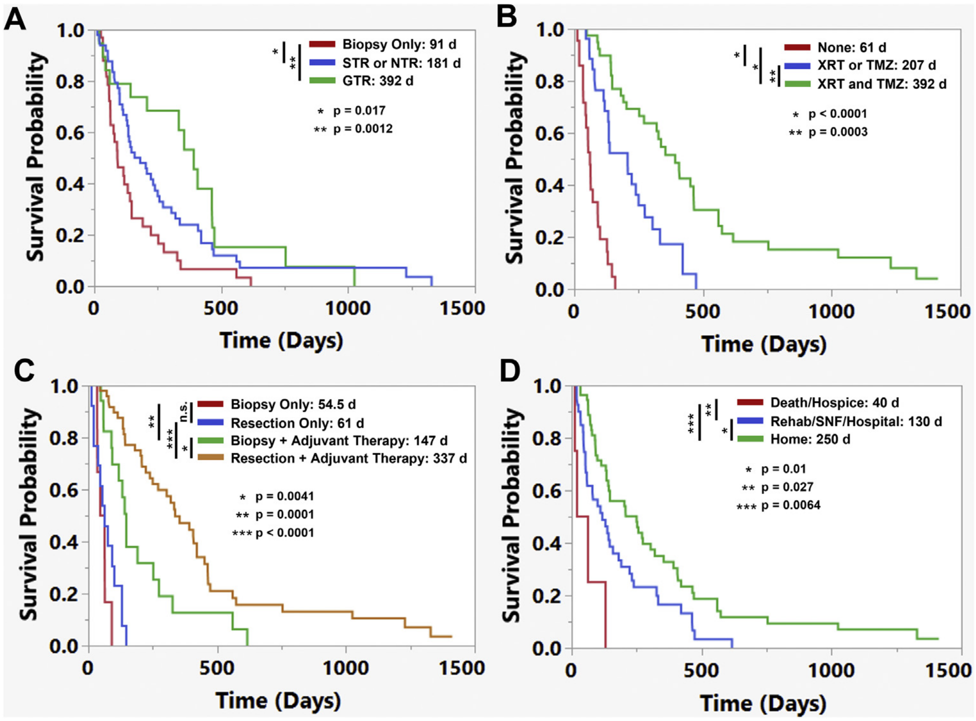

Supplementary Figure 3. Effect of treatment on and association of discharge location with survival. (**A**) Extent of resection influenced survival. The median overall survival from surgery for biopsy, subtotal resection or near total resection, and gross total resection were 91, 181, and 392 days, respectively. (**B**) Adjuvant therapy also influenced survival. The median overall survival for patients receiving no adjuvant therapy, radiotherapy, or temozolomide (i.e., monotherapy), or radiotherapy with concurrent temozolomide were 61, 207, and 392 days, respectively. (**C**) The combination of resection with adjuvant therapy was associated with the greatest duration of survival (337 days) which was significantly longer than that for patients who had undergone biopsy with adjuvant therapy (147 days). No difference was found in survival between patients who had undergone biopsy or resection without additional adjuvant therapy (54.5 vs. 61 days; *P* = NS). (**D**) Discharge location was also significantly associated with overall survival in the cohort.

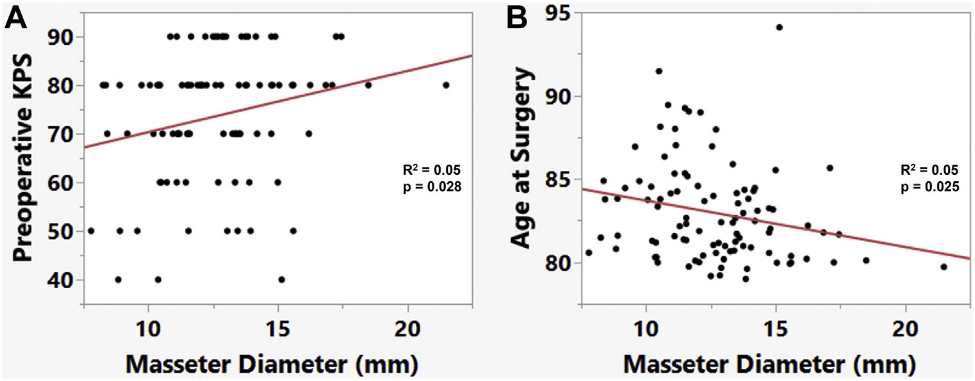

Supplementary Figure 4. Correlation of masseter diameter with patient age at surgery and preoperative Karnofsky performance scale score. (**A**) Masseter diameter correlated weakly but significantly with preoperative Karnofsky performance scale score (R^2 = 0.05; *P* = 0.028). Masseter diameter also correlated weakly but significantly with age at surgery (R^2 = 0.05; *P* = 0.025).
